# A Mobile App for Self-Triage for Pediatric Emergency Patients in Japan: 4 Year Descriptive Epidemiological Study

**DOI:** 10.2196/27581

**Published:** 2021-06-30

**Authors:** Yusuke Katayama, Kosuke Kiyohara, Tomoya Hirose, Tasuku Matsuyama, Kenichiro Ishida, Shunichiro Nakao, Jotaro Tachino, Masahiro Ojima, Tomohiro Noda, Takeyuki Kiguchi, Sumito Hayashida, Tetsuhisa Kitamura, Yasumitsu Mizobata, Takeshi Shimazu

**Affiliations:** 1 Department of Traumatology and Acute Critical Medicine Osaka University Graduate School of Medicine Suita Japan; 2 Department of Food Science Faculty of Home Economics Otsuma Women's University Tokyo Japan; 3 Department of Emergency Medicine Kyoto Prefectural University of Medicine Kyoto Japan; 4 Department of Acute Medicine and Critical Care Center Osaka National Hospital Osaka Japan; 5 Department of Traumatology and Critical Care Medicine Osaka City University Graduate School of Medicine Osaka Japan; 6 Health Services Kyoto University Kyoto Japan; 7 Osaka Municipal Fire Department Osaka Japan; 8 Division of Environmental Medicine and Population Sciences Department of Social and Environmental Medicine Osaka University Graduate School of Medicine Suita Japan

**Keywords:** emergency medicine, self-triage, mobile app, children, telemedicine, app, mobile health, mHealth, epidemiology

## Abstract

**Background:**

When children suffer sudden illness or injury, many parents wonder whether they should go to the hospital immediately or call an ambulance. In 2015, we developed a mobile app that allows parents or guardians to determine the urgency of their child’s condition or call an ambulance and that indicates available hospitals and clinics when their child is suddenly sick or injured by simple selection of the child’s chief complaints and symptoms. However, the effectiveness of medical apps used by the general public has not been well evaluated.

**Objective:**

The purpose of this study was to clarify the use profile of this mobile app based on data usage in the app.

**Methods:**

This study was a descriptive epidemiological study with a 4-year study period running from January 2016 to December 2019. We included cases in which the app was used either by the children themselves or by their parents and other guardians. Cases in which the app was downloaded but never actually used were excluded from this study. Continuous variables are presented as median and IQR, and categorical variables are presented as actual number and percentages.

**Results:**

The app was used during the study period for 59,375 children whose median age was 1 year (IQR 0-3 years). The app was used for 33,874 (57.05%) infants, 16,228 (27.33%) toddlers, 8102 (13.65%) elementary school students, and 1117 (1.88%) junior high school students, with 54 (0.09%) having an unknown status. Furthermore, 31,519 (53.08%) were male and 27,329 (46.03%) were female, with sex being unknown for 527 (0.89%) children. “Sickness” was chosen for 49,101 (78.51%) patients, and “injury, poisoning, foreign, substances and others” was chosen for 13,441 (21.49%). For “sickness,” “fever” was the most commonly selected option (22,773, 36.41%), followed by “cough” (4054, 6.48%), and “nausea/vomiting” (3528, 5.64%), whereas for “injury, poisoning, foreign substances and others,” “head and neck injury” was the most commonly selected option (3887, 6.22%), followed by “face and extremities injury” (1493, 2.39%) and “injury and foreign substances in eyes” (1255, 2.01%).

**Conclusions:**

This study clarified the profile of use of a self-triage app for pediatric emergency patients in Japan.

## Introduction

When children suffer sudden illness or injury, many parents wonder whether they should go to the hospital immediately or call for an ambulance. When they cannot make the decision, they often call for an ambulance, which is one of the reasons why the number of ambulance calls has increased in recent years [1]. Telephone triage services have been introduced to replace various ambulance calls around the world. In the United Kingdom, the National Health Service (NHS) operates a telephone consultation service for people with sudden illness or injury [2]. In Australia, when a patient contacts the emergency dispatch center, secondary telephone triage is conducted after primary triage has taken place [3]. In Japan, telephone triage services for sudden illness and injury have been provided to people mainly in urban areas such as Tokyo and Osaka [4,5]. Osaka Prefecture is one of the largest metropolitan areas in Japan with a population of 8.8 million [6], and the number of ambulance dispatches in this area in 2016 was approximately 600,000 [7]. The Osaka Municipal Fire Department has been operating a telephone triage service (#7119) since 2012 [5]. The number of calls to the telephone triage center in Osaka has been increasing year by year, with the number totaling 284,665 in 2019 [8]. Correspondingly, the use of the telephone triage service has increased, especially during holiday seasons when few medical institutions are open, and this has caused some problems, such as difficulty in connecting to the telephone triage service due to line congestion.

In 2015, we developed a mobile app in Osaka Prefecture that allows parents or guardians to determine the urgency of their child’s condition and call for an ambulance, and which indicates available hospitals and clinics when their child is suddenly sick or injured by having the parents simply select the child’s chief complaints and symptoms. This application has been available in Japan for free download from the Google Play Store and Apple App Store since 2016. The information on available hospitals and clinics that can be displayed in this app is limited to Osaka Prefecture. The widespread use of this mobile app to advise parents and guardians and provide necessary support in the event of sudden illness and injury would enable us to conduct medical services more effectively and to reduce unnecessary ambulance calls and medical costs. In addition, the use of this mobile app may provide assurance to those parents and guardians who have children or dependents with health issues. Clarifying how such a mobile app is used is important for assessing the usefulness of medical mobile apps. Therefore, the purpose of this study was to clarify the profile of the use of this mobile app based on data usage in the app.

## Methods

### Study Design, Population, and Setting

This was a descriptive epidemiological study whose 4-year study period ran from January 2016 to December 2019. In 2015, 17.08 million children were under 15 years old in Japan [6], of whom 1.17 million resided in Osaka Prefecture [6]. We included cases in which the app was used either by the children themselves or by their parents and other guardians in this study. The cases who downloaded the mobile app but never actually used it were excluded from our analysis.

### Telephone Triage Services and Triage Protocol in Osaka Prefecture and Japan

In Japan, there are triage protocols including the Japanese Triage Acuity Scale [9], which is based on the Canadian Triage Acuity Scale, and the Emergency Assessment Protocol [10] established by the Fire and Disaster Management Agency for assessing and triaging emergency patients. Telephone triage services provided in some areas of Japan use the Emergency Assessment Protocol.

The telephone triage service in Osaka Prefecture has been described previously in detail [5,11]. The telephone triage nurses who receive phone calls from people judge the urgency of the chief complaints using software based on the Emergency Assessment Protocol. In Japan, there are 97 different protocols for judging the urgency by chief complaints [10], and the urgency is determined by selecting the signs and symptoms related to the chief complaint. In the mobile app reported here, we selected the 20 protocols from the Emergency Assessment Protocol in Osaka that were the most frequently used in the telephone triage service in Osaka Prefecture for “sickness” and “injury, poisoning, foreign substances and others” (Textbox 1) and modified some of the wording to make it easier for general users to understand.

**Chief complaints listed in the mobile app.**

**Sickness**

- Fever
- Convulsion
- Cough
- Nasal discharge
- Asthmatic attack
- Difficulty breathing
- Rash
- Nausea/vomiting
- Diarrhea
- Stomachache
- Constipation
- Abnormality of the stool
- Ear pain
- Headache
- Crying
- Loss of appetite
- Symptoms of measles
- Other

**Injury, poisoning, foreign substances, and others**

- Head and neck injury
- Face and extremities injury
- Bleeding
- Burn
- Bite wound
- Problem with extremities
- Injury and foreign substances in eyes
- Injury and foreign substances in ears
- Injury and foreign substances in nose
- Other problems in eyes
- Other problems in nose
- Other problems in mouth
- Accidental ingestion of tobacco
- Accidental ingestion of solid material
- Accidental ingestion of liquid material
- Heatstroke
- Other

### Mobile App for Emergency Pediatric Patients

**Figure 1:**
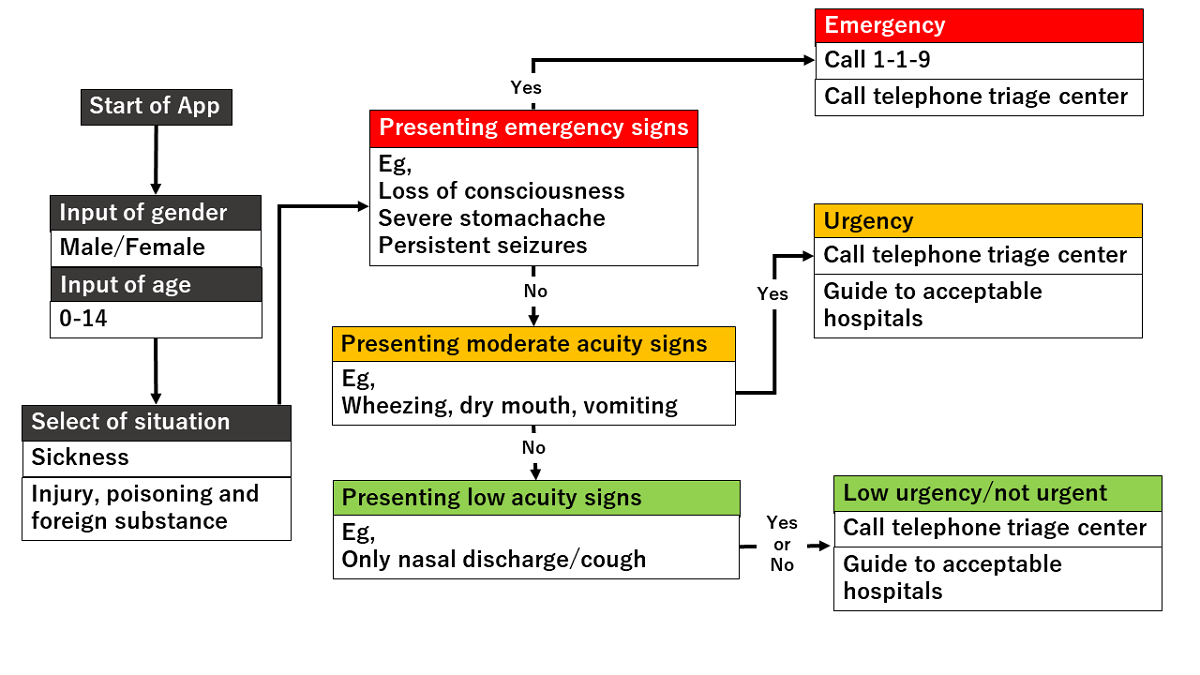
Flowchart of the mobile app.

**Figure 2:**
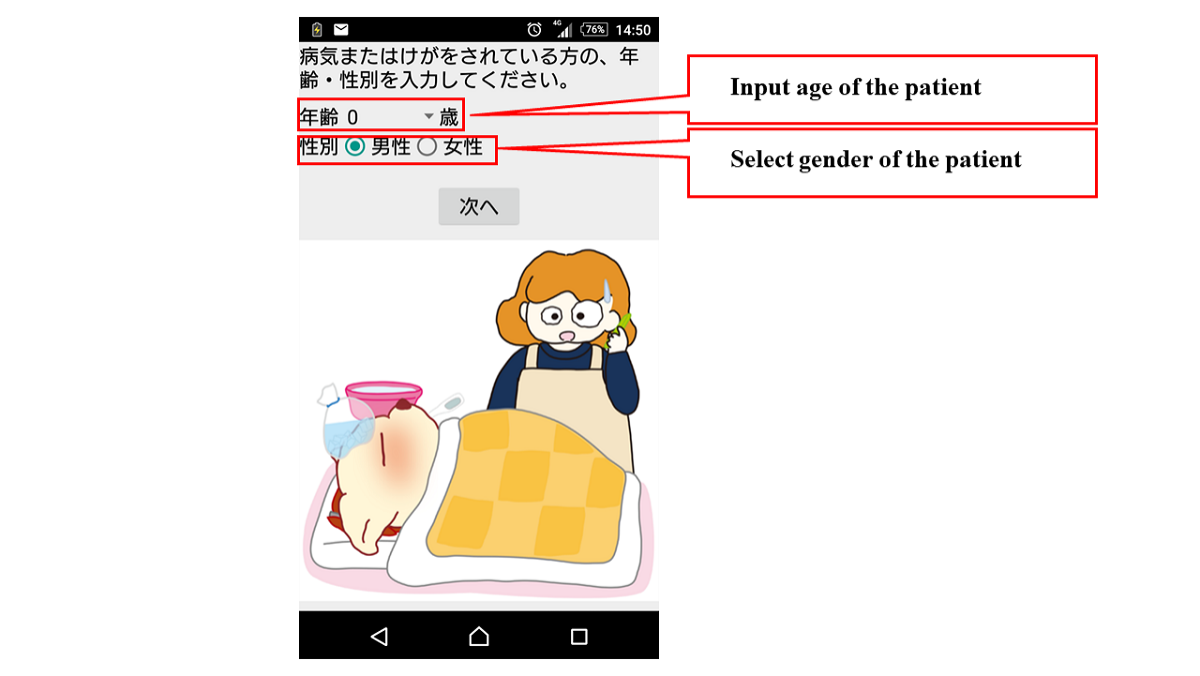
Screenshot of patient input about age and sex.

**Figure 3:**
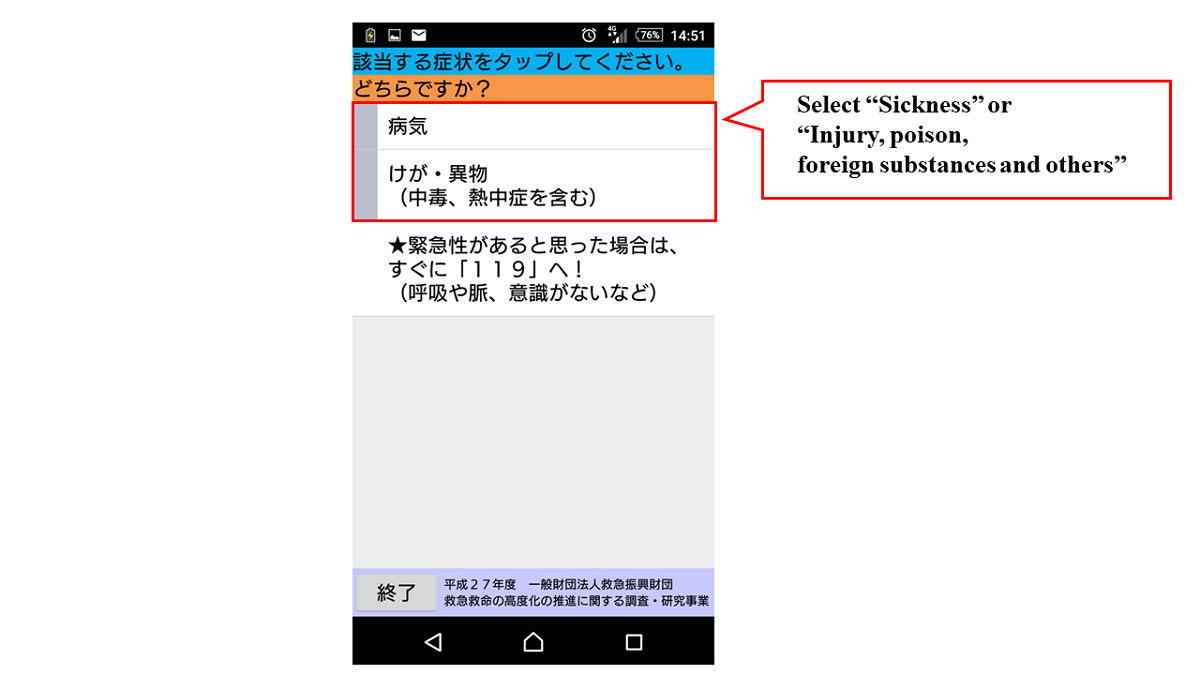
Screenshot of selection about “Sickness” or “Injury, poison, foreign substances and others”.

**Figure 4:**
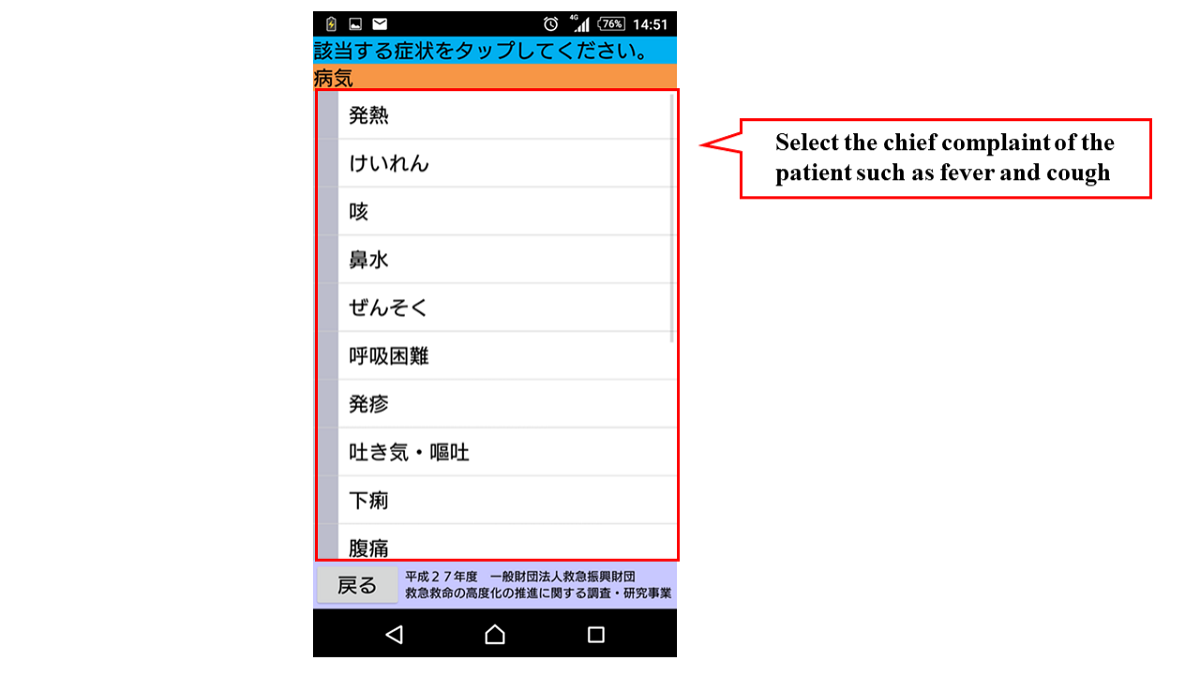
Screenshot of chief complaint selection.

**Figure 5:**
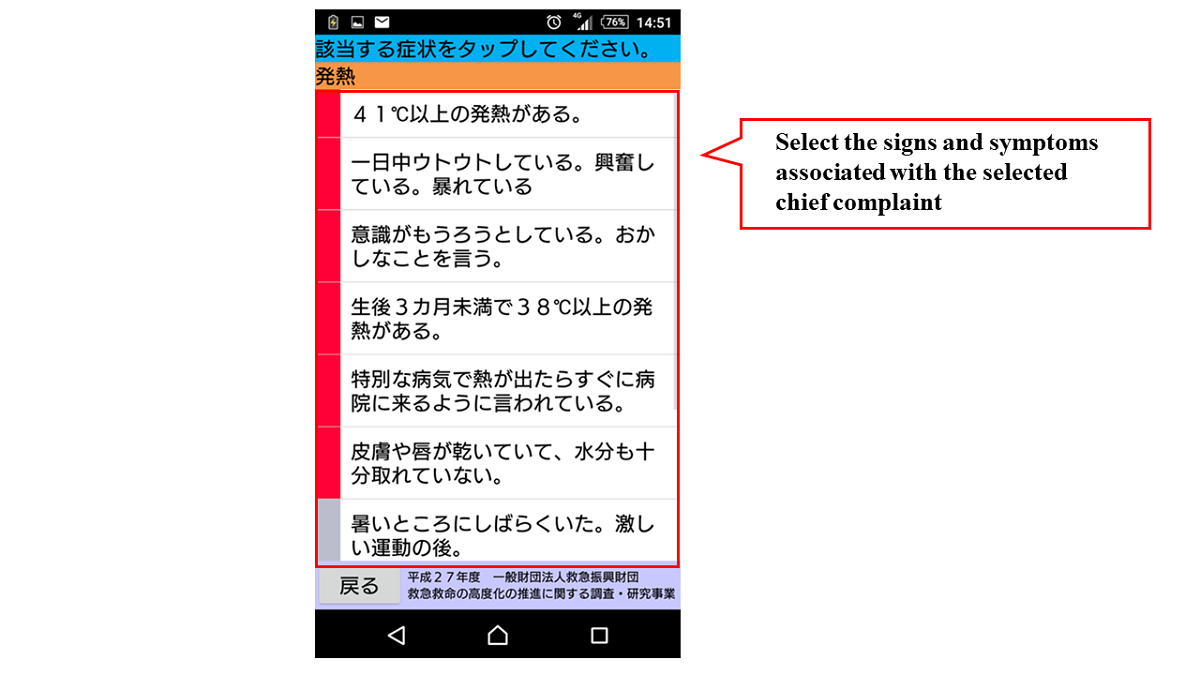
Screenshot about selection of signs and symptoms related to the selected chief complaint.

**Figure 6:**
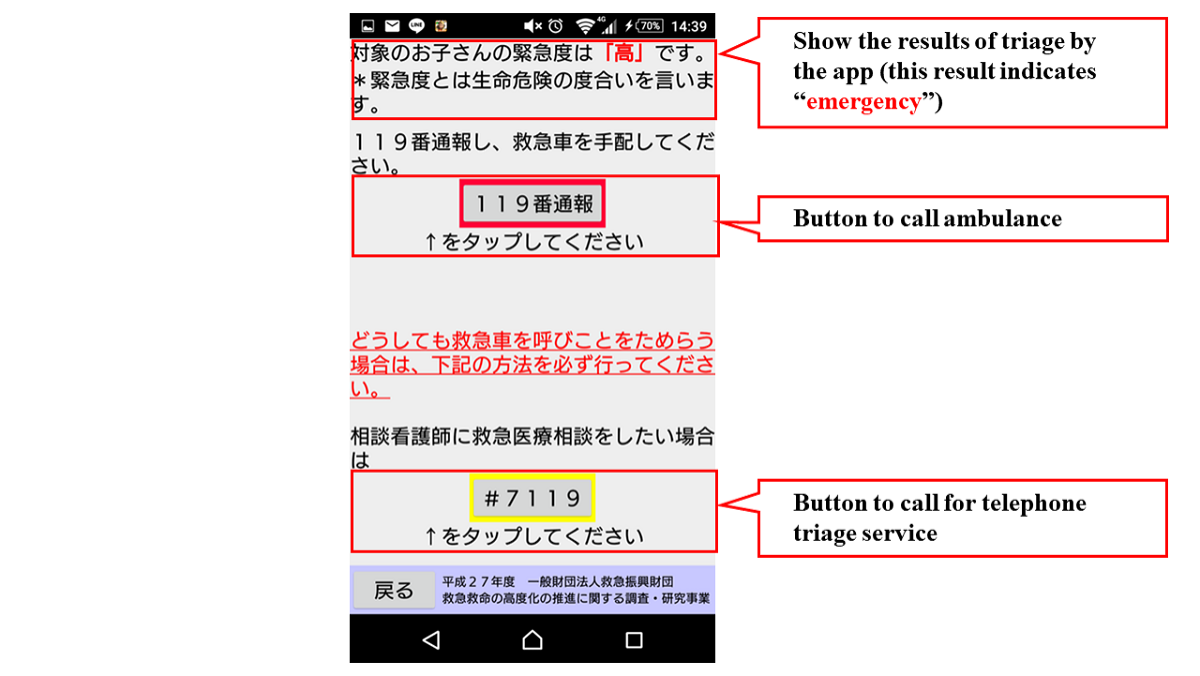
Screenshot of result in emergency cases.

**Figure 7:**
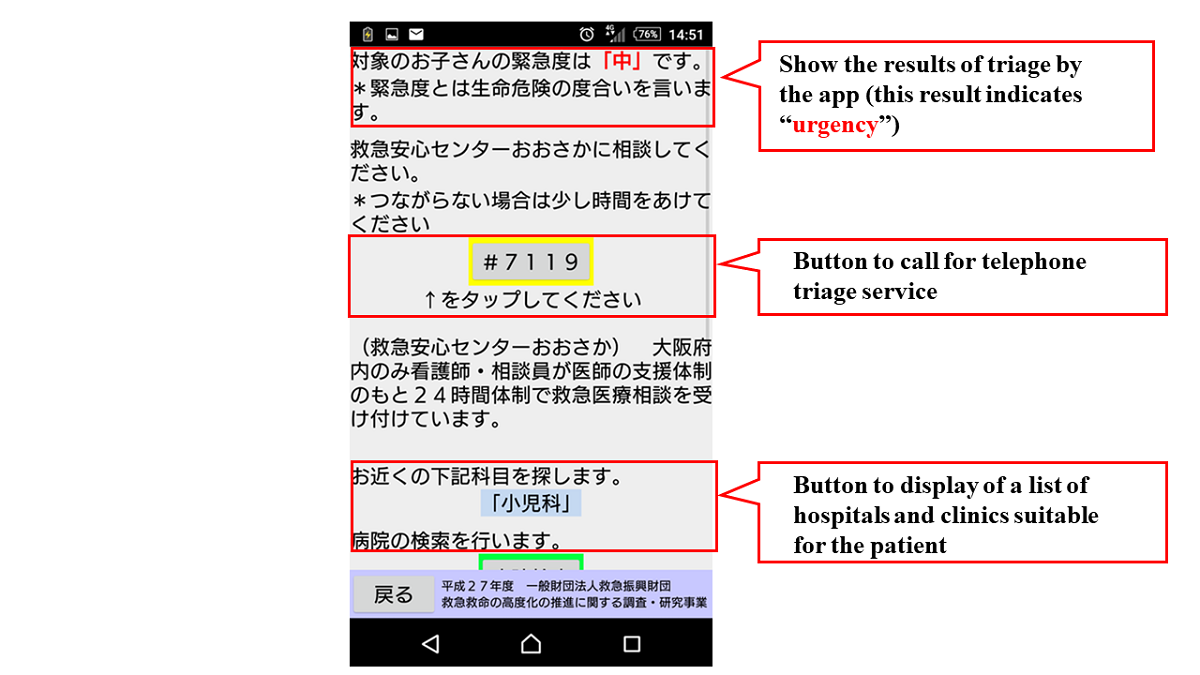
Screenshot of result in urgent cases.

Figure 1 shows the flow chart of the mobile app. The first step in this app is to select the age and gender of the child. Next, the user selects either “sickness” or “injury, poisoning, foreign substances and others.” When either of these is selected, the list of chief complaints shown in Textbox 1 is displayed in the app, and the user selects the relevant chief complaint. For example, if “fever” is selected, relevant signs and symptoms with high urgency such as, “fever of 41℃ or higher,” are displayed in the app. If none of these are selected, relevant signs and symptoms with moderate urgency such as, “decreased urine volume,” are displayed in the app. If none of them apply, the related signs and symptoms corresponding to “low urgency” are further displayed, and the urgency is determined based on the selected signs and symptoms. The app provides emergency medical services, such as the ability to call an ambulance or the telephone triage center and information on available hospitals and clinics. If there is another chief complaint, such as “convulsion,” when “fever” is selected, the app will move to the urgency assessment for the other complaint (Figures 2-7). Only hospitals and clinics in Osaka Prefecture that have agreed to register their information in the app will be displayed as available hospitals and clinics. In addition, the GPS feature of the user’s cellphone also provides a list of hospitals and clinics in order of proximity to the location where the app is being used. During the mobile app development, we asked some mothers for their opinions about the usefulness of this mobile app, and modified the interface of the mobile app. The Android version of this app was released in January 2016, and the iOS version was released in April 2016 for free download. As for the algorithms used in this mobile app, we provided the algorithm for “fever” as one of examples (Table S1, Multimedia Appendix 1).

### Statistical Analysis

Continuous variables are presented as median and IQR, and categorical variables are presented as actual number and percentages. Age groups were categorized as infants (0-1 years), toddlers (2-5 years), elementary school students (6-12 years), and junior high school students (13-15 years). The time of app use was categorized as midnight to 7:59 AM, 8 AM to 3:59 PM, and 4 PM to 11:59 PM. In this study, users who used the app multiple times were counted as a single user. The data used in this study were anonymized, but informed consent was obtained from the users at each occasion of use. This study was approved by the ethics committee of Osaka University Graduate School of Medicine (approval #20313). This manuscript was written based on the Strengthening the Reporting of Observational Studies in Epidemiology (STROBE) statement [12].

## Results

This mobile app was downloaded 24,721 times by December 2019, and we included 22,158 cases for this study, excluding about 2500 users who downloaded this mobile app but did not use it or uninstalled it before using it.

Table 1 shows the characteristics of the children for whom the app was used. The app was used from January 2016 to December 2019 for 59,375 children whose median age was 1 year (IQR 0-3 years). The app was used for 33,874 (57.05%) infants, 16,228 (27.33%) toddlers, 8102 (13.65%) elementary school students, 1117 (1.88%) junior high school students, and 54 (0.09%) children of unknown status. Moreover, 31,519 (53.08%) were male. The number of the children for whom the app was used was 10,105 (17.02%) in 2016, 13,077 (22.02%) in 2017, 17,877 (30.11%) in 2018, and 18,316 (30.85%) in 2019. Regarding the time of day of app use, the app was used between midnight and 7:59 AM by 10,289 patients (17.33%), between 8 AM and 3:59 PM by 20,487 patients (34.50%), and between 4 PM and 11:59 PM by 28,599 patients (48.16%). The app was used by 33,887 patients (57.07%) within Osaka Prefecture and by 5244 patients (8.83%) outside of Osaka Prefecture. Location information was unknown for 20,244 patients (34.10%).

Table 2 shows the clinical characteristics of the patients for whom the app was used. Among the 62,542 chief complaints selected, 49,101 (78.51%) were “sickness” and 13,441 (21.49%) were “injury, poisoning, foreign substances and others.” Among “sickness,” “fever” was the most commonly selected option (22,773, 36.41%), followed by “cough” (4054, 6.48%) and “nausea/vomiting” (3528, 5.64%). Among “injury, poisoning, foreign substances and others,” “head and neck injury” was the most commonly selected option (3887, 6.22%), followed by “face and extremities injury” (1493, 2.39%) and “injury and foreign substances in eyes” (1255, 2.01%).

Of the 4722 patients for whom some kind of emergency medical service was used, the most commonly selected option was “guide to acceptable hospitals and clinics” (3046/4722, 64.51%; Table 3).

**Table 1:** Demographic and geographic characteristics of the patients.

Characteristics			Value, n (%) (N=59,375)
**Age group**			
	Infants (0-1 years old)	33,874 (57.05)	
	Toddlers (2-5 years old)	16,228 (27.33)	
	Elementary school students (6-12 years old)	8102 (13.65)	
	Junior high school students (13-15 years old)	1117 (1.88)	
	Unknown	54 (0.09)	
**Gender**			
	Male	31,519 (53.08)	
	Female	27,329 (46.03)	
	Unknown	527 (0.89)	
**Year**			
	2016	10,105 (17.02)	
	2017	13,077 (22.02)	
	2018	17,877 (30.11)	
	2019	18,316 (30.85)	
**Month**			
	January	4470 (7.53)	
	February	3761 (6.33)	
	March	3917 (6.60)	
	April	4713 (7.94)	
	May	5166 (8.70)	
	June	5324 (8.97)	
	July	5448 (9.18)	
	August	5053 (8.51)	
	September	5299 (8.92)	
	October	4945 (8.33)	
	November	4691 (7.90)	
	December	6588 (11.10)	
**Time of day**			
	Midnight-7:59 AM	10,289 (17.33)	
	8 AM-3:59 PM	20,487 (34.50)	
	4 PM-11:59 PM	28,599 (48.17)	
**Day of the week**			
	Sunday	10,101 (17.01)	
	Monday	8677 (14.61)	
	Tuesday	7817 (13.17)	
	Wednesday	8031 (13.53)	
	Thursday	8764 (14.76)	
	Friday	7477 (12.59)	
	Saturday	8508 (14.33)	
**Area**			
	Inside Osaka Prefecture	33,887 (57.07)	
	Outside Osaka Prefecture	5244 (8.83)	
	Unknown	20,244 (34.10)	

**Table 2:** Clinical characteristics.

Main presenting problem selected on the app		Value, n (%) (N=62,542)
**Sickness**		49,101 (78.51)
	Fever	22,773 (36.41)
	Convulsion	2812 (4.50)
	Cough	4054 (6.48)
	Nasal discharge	2576 (4.12)
	Asthmatic attack	749 (1.20)
	Dyspnea	1414 (2.26)
	Rash	2217 (3.54)
	Nausea/vomiting	3528 (5.64)
	Diarrhea	2034 (3.25)
	Stomachache	959 (1.53)
	Coprostasis	684 (1.09)
	Abnormal stool	560 (0.90)
	Ear pain	793 (1.27)
	Headache	598 (0.96)
	Crying	1182 (1.89)
	Anorexia	472 (0.75)
	Measles symptoms	63 (0.10)
	Symptoms of sicknesses	1633 (2.61)
**Injury, poisoning, foreign substances, and others **		13,441 (21.49)
	Head and neck injury	3887 (6.22)
	Face and extremities injury	1493 (2.39)
	Bleeding	350 (0.56)
	Burn	272 (0.43)
	Bite wound	76 (0.12)
	Problem with extremities	752 (1.20)
	Injury and foreign substances in eyes	1255 (2.01)
	Injury and foreign substances in ears	420 (0.67)
	Injury and foreign substances in nose	358 (0.57)
	Other problems in eyes	121 (0.19)
	Other problems in nose	150 (0.24)
	Other problems in mouth	516 (0.83)
	Accidental ingestion of tobacco	622 (0.99)
	Accidental ingestion of solid material	848 (1.36)
	Accidental ingestion of liquid material	235 (0.38)
	Heatstroke	1073 (1.72)
	Other symptoms of injury, poisoning, foreign substances and others	1013 (1.62)

**Table 3:** Clinical characteristics.

Users’ input	Value, n (%) (n=4722)
Call for ambulance	33 (0.7)
Call for telephone triage service	422 (8.9)
Call for medical consultation service of children	3 (0.1)
Guide to acceptable hospitals and clinics	3046 (64.5)
Observation	1218 (25.8)

## Discussion

### Principal Results

This study is the first to describe the profile of use of an emergency medical app available to the general public that guides them in the event of a child’s sudden illness or injury. The app was most frequently used for infants, and it was used most frequently between 4 PM and 11:59 PM and on Sundays. In addition, about half of the uses were in Osaka Prefecture. The most common medical advice selected was “guide to acceptable hospitals and clinics.” This study would be useful in the development of medical apps that can be used on mobile devices for nonmedical personnel.

We previously described the profile of patients who were transported by ambulance after telephone triage in Osaka Prefecture [5]. The most common time of day for both telephone triage and use of this app was in the evening. In Japan, some nighttime emergency clinics provide primary emergency care after many general clinics close in the evening. It is likely that when a child suddenly becomes sick or injured after clinics hours, parents use the telephone triage service or the app to decide whether they should visit a nighttime emergency clinic or emergency department immediately, call for an ambulance, or wait until the next day. This result was similar to the finding of a study of telenursing and an advice line in Australia [13]. In this study, about 57% of the users used this mobile app within Osaka Prefecture. The difference in the number of users within and outside of Osaka Prefecture might have been influenced not only by the available services, such as visiting medical institutions, but also by the public activities of the government. In Osaka Prefecture, the fire departments have not only publicized the mobile app through advertisements on trains and posters but also conducted activities to introduce the mobile app at events of basic life support for citizens. These government activities might have helped to increase awareness about this mobile app among citizens. In order to provide health services to citizens via mobile apps, it is essential for governments to repeatedly promote and educate the public.

The app assessed here not only judges urgency based on the selection of signs and symptoms but also provides medical help, such as ambulance calls and a guide to appropriate hospitals and clinics based on the results of the judgment. As a result, the number of users of this app has increased over the years, which indicates that not only the interface of this app but also the services it provides have been widely accepted by many users. There are several models that explain people’s acceptance of new technologies. Venkatesh and colleagues [14] stated that the people’s acceptance of new technologies is associated with 4 factors: performance expectancy, effort expectancy, social influence, and facilitating conditions (the Unified Theory of Acceptance and Use of Technology [UTAUT]). For example, performance expectancy is defined as the degree to which an individual believes that using the system will help him or her to attain gains in job performance. The emergency room system in the United States is not common in Japan. Therefore, when people suffer from sudden illness or injury, they have to use the phone or the internet to find hospitals and clinics by themselves. This mobile app assesses the urgency level of their selected symptoms and provides information on appropriate hospitals and clinics that can treat the patient. Hence, as it is more convenient to search than the traditional search for hospitals or clinics, people’s performance expectancy is applicable to this mobile app. In addition, the effort expectancy is defined as the degree of simplicity associated with the use of a system. During the development of this mobile app, we asked a few mothers for their opinions about the usefulness of this mobile app, and modified the interface of the mobile app. Thus, although this mobile app was not always developed based on behavioral theories like UTAUT, some elements from these theories may be applicable.

### Comparison With Prior Work

In this study, the number of ambulance calls made via the mobile app was low. In a previous study by Morimura and colleagues [15], 12.9% of patients who used the telephone triage services connected the phone to the ambulance dispatch center. The following may explain the discrepancy between this previous study and our own. First, Morimura et al’s study included not only children but also adults and older adults, whereas we included only children in this study. Therefore, children might have been taken to a medical facility by their parents or guardians instead of by ambulance even when the urgency level of their symptoms was high. Second, the parents and guardians who used this mobile app might have only wanted to know the degree of urgency or whether or not they should visit a medical facility immediately. Indeed, only 7.95% (4722/59,375) of the population who used the mobile app used medical services, such as an ambulance call or an information search on medical institutions.

Many mobile apps for medical personnel have been developed in the field of pediatric emergency medicine. For instance, another study on medical app use in Irish pediatric emergency departments reported that one-third of medical facilities and about half of the medical personnel had medical apps installed on their personal mobile devices, with the most adopted app being the British National Formulary app developed by the NHS [16]. However, the effectiveness of medical apps used by the general public has not been well evaluated. In a review of 175 studies on medical apps that support self-management for the general public, only 30.3% of the apps were publicly available from app stores, the number of study participants was small (median number of study participants 31; IQR 11.0-29.2; maximum 11,690), and clinical outcomes were evaluated in only 36 studies (20.6%) [17]. Another study comparing the effectiveness of an app, book, and video program on child health for parents visiting the emergency department reported that the app was not widely used, and the parents did not recommend the app to others [18]. For an app to be widely accepted by the general public, the effectiveness of the service provided by the app and an easy-to-use interface for users are essential. The app assessed here not only judges urgency based on the selection of signs and symptoms but also provides medical help through services like ambulance calls and guidance to appropriate hospitals and clinics based on the results of the assessment. As a result, the number of users of this app has increased over the years, which indicates that both the interface and services of the app have been widely accepted by many users. In addition, the number of app users is larger than that in previous studies, and thus, the general validity may also be high.

The purpose of this study was to determine the profile of use for this mobile app based on data usage in the app as mentioned in the Introduction; however, we did not evaluate the effectiveness of this mobile app. The app may have effects on the prognosis of patients as well as various other aspects, such as medical costs and the number of unnecessary ambulance calls. We are further planning to verify the efficacy of our mobile app through long-term monitoring or via a random interview survey to clarify other outcomes for users in the future, such as satisfaction of user experience.

### Limitations

This study has several limitations. First, as the data entered into the app only came from users, it is possible that the data entered did not accurately reflect the patient’s condition. Second, there were no data on the prognosis of any of the patients. Although the cooperation of users is essential for investigating the prognosis of the patients, there are legal problems in follow-up investigation. Moreover, this study did not ask for user demographics, and it was unclear whether a given user of the app was a parent or guardian. Third, compared to telephone triage, validation of the user’s own triage using the app is problematic. We plan to examine this limitation in the future. Fourth, since the mobile app is, unfortunately, not yet sufficiently widespread in our target region, we must further make efforts to disseminate this app so that we can evaluate the effect of this app on ambulance calls and medical costs. Fifth, it was unclear to us whether users wanted to just try out the app or if they really wanted to use it. Sixth, demographics, including whether app users were adults, guardians, or the children themselves as well as other detailed information, such as average usage time and triage time, were not available in this study. Finally, we did not verify the validity of the of urgency assessment provided by this mobile app, which is something which should be determined in a future study.

### Conclusions

In this study, we clarified the use profile of a self-triage app for pediatric emergency patients in Japan.

## Appendix

Multimedia Appendix 1 Table S1. The algorithm for “fever” in the mobile app.

## Abbreviations

NHS: National Health Service
STROBE: Strengthening the Reporting of Observational Studies in Epidemiology
UTAUT: Unified Theory of Acceptance and Use of Technology
